# Selective Survival and Maturation of Adult-Born Dentate Granule Cells Expressing the Immediate Early Gene Arc/Arg3.1

**DOI:** 10.1371/journal.pone.0004885

**Published:** 2009-03-17

**Authors:** Sjoukje D. Kuipers, Adrian Tiron, Jonathan Soule, Elhoucine Messaoudi, Andrea Trentani, Clive R. Bramham

**Affiliations:** Department of Biomedicine and Bergen Mental Health Research Center, University of Bergen, Bergen, Norway; Universidade Federal do Rio de Janeiro (UFRJ), Instituto de Biofísica da UFRJ, Brazil

## Abstract

Progenitor cells in the adult dentate gyrus provide a constant supply of neuronal precursors, yet only a small fraction of these cells survive and develop into mature dentate granule cells (DGCs). A major challenge of current research is thus to understand the stringent selection process that governs the maturation and functional integration of adult-born DGCs. In mature DGCs, high-frequency stimulation (HFS) of the perforant path input elicits robust expression of the immediate early gene Arc/Arg3.1, trafficking of its mRNA to dendrites, and local synthesis of the protein necessary for consolidation of long-term potentiation (LTP). Given the synaptic commitment inherent in LTP consolidation, we considered that HFS-evoked expression of Arc could be used to timemap the functional integration of newborn DGCs. Dividing cells were birthmarked by BrdU-labeling at 1, 7, 14, 21, or 28 days prior to induction of LTP and expression of Arc was examined by confocal microscopy. Contrary to expectation, LTP did not induce Arc expression in newborn cells at any age, suggesting they might be refractory to synaptically-evoked Arc expression for at least one month. Importantly, however, spontaneous expression of Arc was detected in BrdU-labeled cells and strongly associated with the survival and maturation of NeuN-positive DGCs. Moreover, Arc expression at the earliest ages (1 and 7 days), clearly precedes the formation of glutamatergic synapses on new neurons. These results suggest an unexpected early role for Arc in adult-born DGCs, distinct from its functions in LTP, LTD, and homeostatic synaptic plasticity.

## Introduction

New neurons continue to be generated in the adult mammalian brain. Neurogenesis, which occurs solely in the subventricular zone (SVZ) of the olfactory bulb and the dentate gyrus of the hippocampal formation, encompasses the proliferation of neural stem and progenitor cells and the differentiation, migration and eventual incorporation of neurons into the pre-existing circuitry [1], [2]. In the dentate gyrus, new dentate granule cells (DGCs) are generated throughout life from precursor cells residing in the subgranular zone (SGZ) [3], [4]. Although this form of neural plasticity is recognized to contribute to hippocampal-dependent memory function [5]–[10], relatively little is known regarding the functional integration of newly born cells into pre-existing neuronal networks.

Ample evidence supports a role for activity-dependent synaptic plasticity in hippocampal-dependent learning and memory [11], [12], with recent work specifically implicating long-term potentiation (LTP)-like mechanisms. The formation of stable protein synthesis-dependent LTP in adult DGCs is a tightly controlled process requiring upregulation of the immediate early gene (IEG) Arc (activity-regulated cytoskeletal-associated protein)/Arg3.1 (activity-regulated gene 3.1 protein homolog) [13]. Arc mRNA is rapidly induced by high-frequency stimulation (HFS) of perforant path input, trafficked to granule cell dendrites and locally translated [14]–[16]. A recent study employing Arc antisense oligodeoxynucleotides showed that sustained Arc synthesis during a critical time window after LTP induction is necessary for LTP consolidation and growth of the F-actin cytoskeleton that is thought to underlie stable changes in spine morphology [17]–[20]. There is similarly a wealth of evidence implicating Arc expression patterns in hippocampal dependent and independent forms of long-term memory [21]–[24]. Given the heavy energy demands of new protein synthesis and the synaptic commitment inherent in LTP consolidation, we considered that synaptically-evoked Arc expression could be used to map the timecourse of functional maturation of adult-born DGCs, while also gaining insight into the underlying mechanisms.

To this end, newly dividing cells were birthmarked by injection of the thymidine analogue BrdU, and LTP was induced 1, 7, 14, 21, and 28 days thereafter. Surprisingly, although Arc was sharply upregulated in the pre-existing granule cell population, LTP-inducing stimulation did not enhance Arc expression in newborn cells of any age. Instead, we found expression of Arc protein in the absence of LTP induction. Furthermore, newborn neurons positive for Arc expression exhibited selective survival and maturation as NeuN-positive DGCs in the granule cell layer. This demonstration of Arc expression at the earliest ages (1–7 days) suggests a function for Arc in newborn cells that might be distinct from its role in LTP consolidation in the pre-existing granule cell population.

## Materials and Methods

### Animals

Male Sprague Dawley rats (Møllegårds Avlslaboratorium, Denmark; *n* = 40: 250–350 g and all between 10–12 weeks of age at the time of BrdU injection) were individually housed with ad libitum access to food and water under climate-controlled conditions (23°±0.5°C). Rats were maintained on a 12-h light/dark cycle (light on at 07:00) and received at least 7 days to acclimatize to their new environmental conditions prior to onset of the experiment. This investigation, designed to minimize the number of animals and suffering, was carried out in accordance with the Norwegian Regulation on Animal Experimentation, the European Convention for the Protection of Vertebrate Animals used for scientific purposes and the guidelines of the Norwegian Animal Research Authority.

### BrdU Injections

**Figure 1:**
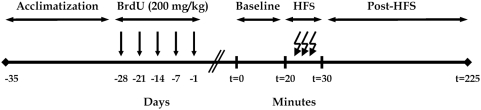
Schematic representation of the experimental design. A timeline illustrating the sequence of events comprising the experimental setup is shown. Experiments commenced after one week of acclimatization. LTP was induced to probe for Arc expression and BrdU (200 mg/kg; i.p.) was administered 1, 7, 14, 21, or 28 days prior, to time-stamp new neurons in progressive stages of their functional development. After a 20 min baseline, LTP was induced by spaced stimulation of HFS (4 sessions; 400 Hz, 8 pulses, 5 min intervals). Animals were killed and tissue was collected 195 min post recording, to proceed with histological analysis of maturation across the 28-day window. Arc expression was examined to investigate functional network activity of adult-born granule cells in the hippocampus.

A single 5-bromo-2′-deoxyuridine injection (BrdU; 200 mg/kg; Sigma) was given intraperitoneally (i.p.) 1, 7, 14, 21, or 28 days prior to LTP induction. This design allowed us to ascertain Arc expression in new neurons throughout their proliferative (24 h) phase to their long-term survival (4 weeks) (Fig. 1).

### Electrophysiology

**Figure 2:**
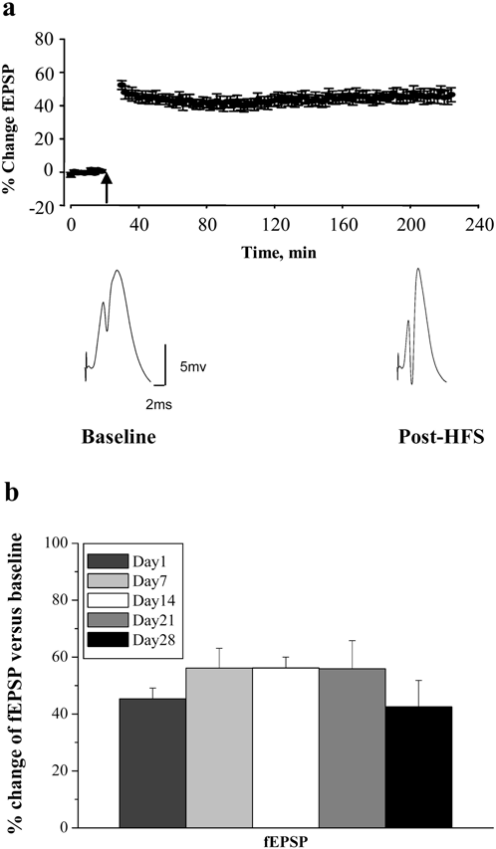
Stable LTP in BrdU-injected rats. a, Time course plot illustrates changes in the medial perforant path-evoked fEPSP slope expressed in percentage of baseline. Values are means±SEM. HFS is indicated by the arrow. Average field potential traces (10 sweeps) collected at the end of baseline (Baseline) and at the end of recording (Post-HFS) are shown below. Calibration: 5 mV, 2 ms. b, Magnitude of fEPSP slope changes was equal across all groups. n = 8 per group.

LTP was induced in the DG, using methodology previously described [25], [26]. Rats were anesthetized with urethane (1.5 g/kg i.p.) and placed in a stereotaxic apparatus. Rectal temperature was maintained at 36.5°C using a servo-heating pad. Electrodes were placed for selective unilateral stimulation of the medial perforant path and recording of evoked field potentials from the dentate gyrus. Stereotaxic coordinates relative to Bregma were 3.9 mm posterior, 2.3 mm lateral for recording and 7.9 mm posterior, 4.2 mm lateral for stimulation. A bipolar stimulating electrode was placed into the dorsomedial angular bundle for stimulating the medial perforant path. After making a slit in the dura, a Teflon-coated stainless steel wire recording electrode was slowly lowered into the dorsal hippocampus until positive field excitatory postsynaptic potential (fEPSP) of maximum slope was obtained in the dentate hilus. Biphasic rectangular pulses of 150 µs duration were applied every 30 sec throughout the experiment, using an intensity set to elicit a population spike amplitude of 30% of the maximal response. After 20 minutes of stable baseline recording, LTP was induced using a 400 Hz protocol that consisted of eight pulses, repeated four times, at 10 sec intervals. Three sessions of HFS were given at intervals of 5 minutes. Signals from the dentate hilus were amplified, filtered (1 Hz to 10 kHz), and digitized (25 kHz). Acquisition and analysis of field potentials was accomplished using DataWave Technologies (Longmont, CO) WorkBench software. Responses were normalized to baseline and statistics were based on values obtained during the last 5 minutes of recording (Fig. 2a,b). All animals included in the study exhibited stable fEPSP slope increases of at least 25% at the end of LTP recording.

### Tissue collection

Subsequently, 195 minutes after completing the HFS protocol, rats were transcardially perfused with 4% paraformaldehyde solution in 0.1 M sodium phosphate buffer (pH 7.4). Brains were removed and post-fixed in the same solution overnight at 4°C, before being transferred to a sodium phosphate buffer (NaPBS 0.02 M, pH 7.4) and stored at 4°C. Following cryoprotection of the brains by overnight immersion in a 30% glucose solution, 30 µm coronal serial sections were prepared on a cryostat microtome. Sections were collected in NaPBS with sodium azide and stored at 4°C. Immunohistochemical stainings were performed on the same animals using parallel sets of coronal sections.

### In Situ Hybridization

**Figure 3:**
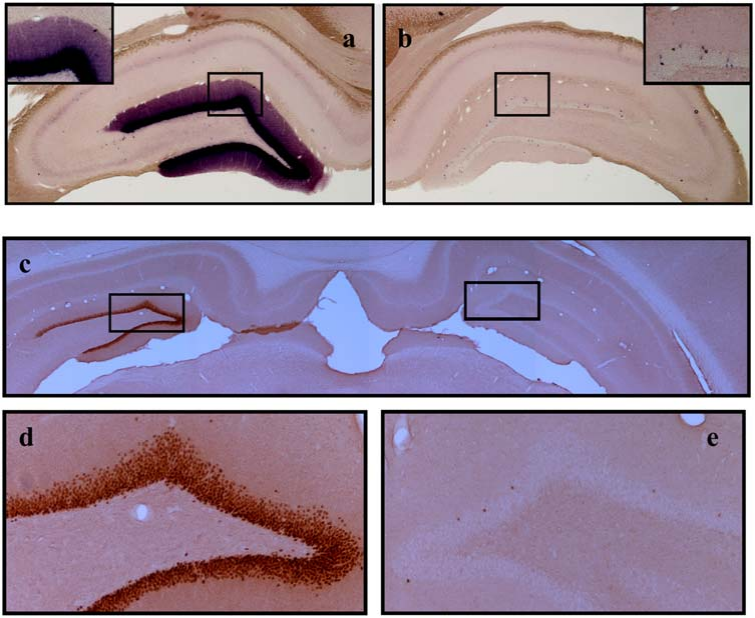
Arc mRNA and protein expression after LTP induction in the dentate gyrus. a–b, Arc *in situ* hybridization (ISH) and c–e immunohistochemistry (IHC) were performed 195 min after high frequency stimulation of the medial perforant pathway. Stimulated hemispheres received 4 sessions of 400 Hz bursts separated by 5 min intervals and control hemispheres received no electrode implantation or stimulation. a, Coronal sections processed for ISH using a digoxigenin-labeled riboprobe show robust upregulation of Arc mRNA in granule cell somata and dendrites on the stimulated side. b, Arc mRNA was clearly less on the contralateral side. The inserts show high-magnification images of Arc ISH in the corresponding stimulated and non-stimulated dentate gyrus. c, The representative photomicrograph of Arc IHC illustrates rapid and sustained elevation of Arc protein after HFS on the stimulated side relative to the non-stimulated controlateral control. d. High magnification images taken from the stimulated and e, non-stimulated dentate gyrus above. All panels are representative images based on 8 animals in each treatment group. Images were obtained from the mid-dorsal dentate gyrus within ∼300 µm of the recording site.

RNA probes were prepared from a cDNA insert matching the first 2975 nucleotides of the Arc mRNA (GenBank accession number NM_019361) cloned into the pCRII-TOPO vector (Invitrogen, Oslo, Norway). Antisense and sense probes were transcribed from linearized plasmids using T7 and SP6 polymerase in the presence of digoxigenin (DIG) labeling mix according to instructions of the manufacturer (Roche, Indianapolis, IN). Floating sections were placed in PBS for 5 min, permeabilized with proteinase K (10 µg/ml) for 5 min at 37°C, and postfixed (5 min with 4% paraformaldehyde/PBS). After fixation, sections were treated with 0.25% acetic anhydride in 0.1 M triethanolamine, pH 8, for 10 min, washed twice in 2×SSC, and placed for 10 min in a prehybridization buffer. Probes were applied to the sections and hybridization was performed in a humidified chamber at 60°C for at least 16 hours. Sections were washed twice with 2×SSC at RT for 30 min, once with 50% formamide in 2×SSC at 65°C, rinsed in 2×SSC at 37°C, incubated with 20 µg/ml RNase A at 37°C for 30 min, and incubated in RNase A buffer at 65°C for 30 min. After blocking in 2% blocking reagent for 1 hour at RT, alkaline phosphatase-coupled anti-DIG antibody (1∶2000; Roche) was applied for another hour at RT. Visualization was done with the chromogenic substrates nitroblue-tetrazolium-chloride and 5-bromo-4-chlor-indolyl-phosphate (Roche). Hybridization using the Arc sense probe remained unstained and served as a control. Pictures were taken on a Nikon (Tokyo, Japan) Eclipse 80i microscope coupled to a Nikon DS-5M camera (Fig. 3a,b).

### Immunohistochemistry

For all stainings (unless otherwise specified) every sixth section throughout the rostral/caudal extent of the hippocampus (Bregma −2 to −6) was collected and coded before processing for immunohistochemistry to ensure objectivity. Stainings were performed on free-floating sections under continuous agitation.

#### Arc immunohistochemistry

Sections were preincubated in 0.3% H_2_O_2_ for 15 min to reduce endogenous peroxidase activity, before being incubated in the primary polyclonal rabbit anti-Arc antibody raised against amino acids 1–300 of the Arc protein (H300; SantaCruz Biotechnology, Santa Cruz CA, sc-15325; 1∶500 dilution in NaPBS 0.02 M, pH 7.4) for 60–72 h at 4°C. Subsequently, sections were washed with NaPBS and incubated at room temperature with biotinylated donkey anti-rabbit IgG (Amersham Biosciences, Sunnyvale, CA; 1∶500). To confirm specificity of results obtained with the applied antibody, comparative staining was performed using an alternative mouse monoclonal antibody for Arc raised against amino acids 264–385 of the Arc protein (BD Biosciences; San Jose, CA; # 612603) (Fig. 3c–e). Furthermore, a rabbit polyclonal antibody for Egr-1 (a.k.a. Zif268) (Santa Cruz Biotechnology, Santa Cruz CA, sc-110; 1∶200) was included as an additional positive control for LTP-induced IEG expression in new neurons.

#### BrdU immunohistochemistry

**Figure 4:**
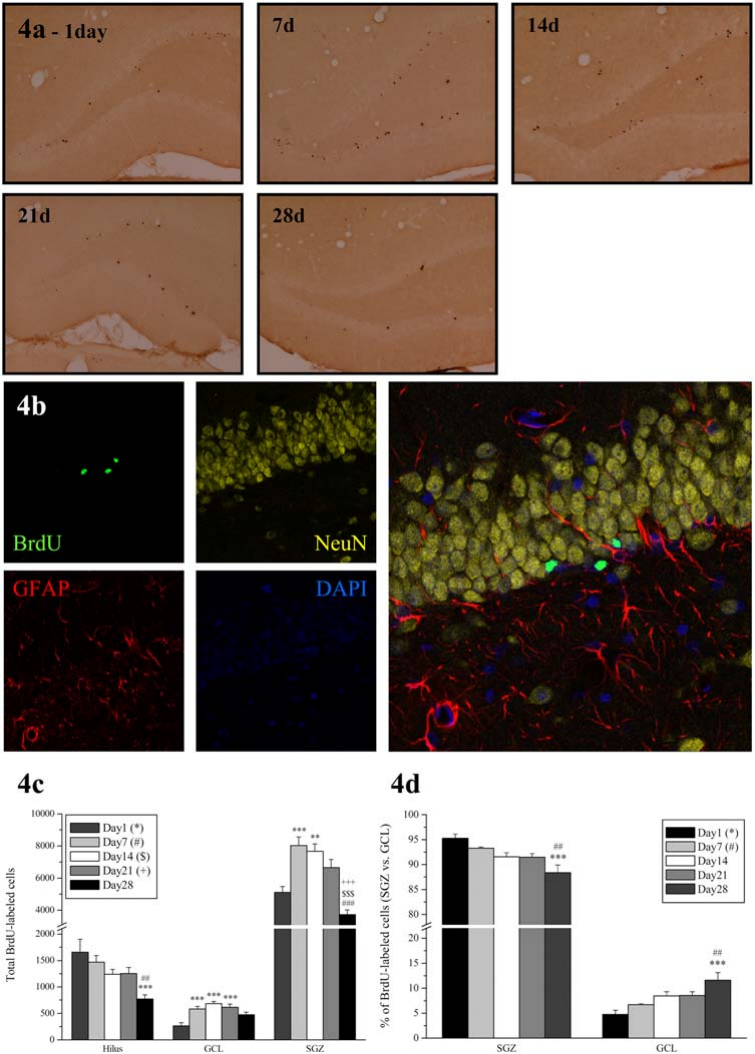
Total BrdU-labeled cells in the dentate gyrus. a, Representative photomicrographs depicting the time course of immunohistochemical BrdU labelling of new DGCs across the age groups. b, Confocal image illustrating immunofluorescent multi-labeling of BrdU-NeuN-GFAP-Dapi taken from an animal of the 1d group. Observations reveal that BrdU cells (green) are located primarily along the SGZ. While initially negative for NeuN expression at 1 day the majority of new cells become neuronal by 28 days (BrdU-NeuN = 83.9%) c, Graph illustrating the average total number of BrdU-labeled cells in the hippocampal granule cell layer (GCL), subgranular zone (SGZ) and hilus per group (n = 8) ±SEM. d, The total number of BrdU-labeled cells in SGZ and GCL depicted as a percentage of the total number of new cells in both regions. The *, #, $, + symbols represents significant effects compared to day 1, 7, 14, 21 respectively. One, two or three symbols represent p<0.05, p<0.005, p<0.0005 respectively.

Peroxidase BrdU labeling was performed as previously described [3], [27]. In short, BrdU-labeling requires the following pretreatment steps: DNA denaturation (50% formamide/2×SSC (pH 7.0), 65°C, 120 min), and acidification (2 M HCl, 30 min). Primary antibody was a monoclonal rat anti-BrdU (Oxford Biotechnology, OBT0030CX; 1∶800 dilution in NaPBS 0.02 M, pH 7.4) while secondary antibody was a biotin-SP-conjugated donkey-anti-rat (Jackson ImmunoResearch Laboratories, Inc., West Grove, PA, USA; 1∶400 dilution in NaPBS 0.02 M, pH 7.4). Sections were subsequently incubated with ABC (Vector ABC kit, Vector Laboratories, Burlingame, CA, USA) and after another wash, reaction products were visualized by adding diaminobenzidine as chromogen and 1% H_2_O_2_ for 15 min (Arc) or 30 min (BrdU). Finally, sections were washed, mounted on slides, dehydrated and coverslipped with DPX (Fig. 4a).

#### Immunohistofluorescent multi-labeling

**Figure 5:**
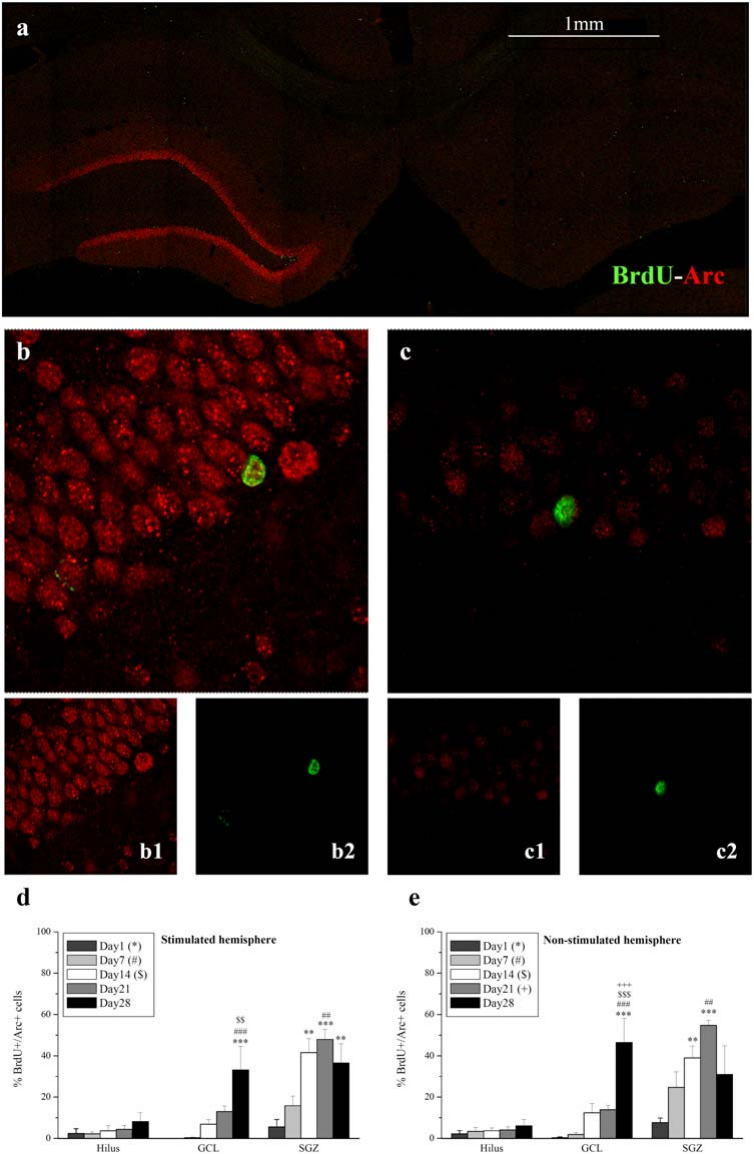
Percentages of BrdU^+^/Arc^+^ cells in the dentate gyrus illustrate constitutive expression of Arc in DGCs. a, Representative confocal image of sections (control and LTP sides) stained for BrdU (green) with Arc (red) from an animal injected with BrdU prior to LTP induction. The photomicrograph reveals strong LTP-induction of Arc on the stimulated side. 40× magnification image of a b,b1,b2 stimulated and c,c1,c2, control DG. Images of Arc and BrdU depicted by separate channels reveal colocalization in the same DGCs. d, Graphs illustrating the percentages of BrdU+ cells, positively labelled for Arc, in the hippocampal subregions on the stimulated hemisphere and e, non-stimulated hemisphere, reveal that young cells are surprisingly refractory to HFS-induced Arc expression across all ages. The *, #, $, + symbols represents significant effects compared to day 1, 7, 14, 21 respectively. One, two or three symbols represent p<0.05, p<0.005, p<0.0005 respectively.

For immunohistofluorescent multi-labeling of BrdU, one in 12 sections were denaturated, followed by 60–72 hours incubation in monoclonal rat anti-BrdU (Oxford Biotechnology, OBT0030CX; 1∶300 dilution in NaPBS 0.02 M, pH 7.4) together with polyclonal rabbit anti-Arc H300 (SantaCruz Biotechnology, Santa Cruz CA, sc-15325; 1∶100) and monoclonal mouse anti-NeuN (Chemicon International; MAB377; 1∶200) or the latter with polyclonal rabbit anti-GFAP (DakoCytomation; Z0334; 1∶500). Sections were then incubated in Alexa-488 donkey anti-rat (Molecular Probes; A21208; 1∶200) followed by Alexa-555 donkey anti-mouse (Molecular Probes; A31570; 1∶200) and Alexa-647 donkey anti-rabbit (Molecular Probes; A31573; 1∶200) antisera (Fig. 4b, 5a–c).

### Image Analysis and Quantification

Quantification of BrdU-labeling was conducted using a modified unbiased stereology protocol [27], [28]. All BrdU-labeled cells in the granule cell layer (GCL), subgranular zone (SGZ) and hilus were counted (at 100×) regardless of size or shape using a Nikon Eclipse 80i microscope (Tokyo, Japan) coupled to a Nikon DS-5M camera. Cells were considered as being in the subgranular zone if they were located in or touching the subgranular zone. Cells that were located more than two cell widths away from the subgranular zone were counted as in the hilus. To enable counting of cell clusters, cells were examined under 400× and 1000× magnifications. Quantification was conducted bilaterally and the total number of BrdU-labeled cells was estimated by multiplying the number of cells counted in every sixth section by 6 and reported as the total number of BrdU-labeled cells (mean and SE) [27], [28].

For multi-labeling, percentages of BrdU-labeled nuclei coexpressing Arc and NeuN, or GFAP were determined by analyzing 50–100 randomly selected BrdU-labeled nuclei throughout the GCL and SGZ using a Leica TCS SP2 AOBS confocal microscope (Leica Microsystems, Heidelberg GmbH). Absolute cell numbers of BrdU+/Arc+ and BrdU+/Arc− cells were calculated by multiplying the total number of BrdU+ cells by their estimated percentages. Care was taken to verify double-labeling and control for false positives by analyzing BrdU-positive nuclei in their *z*-axis and rotating them in orthogonal x-y planes using a 63× oil objective (1 µm steps). To exclude potential cross-bleeding between detection channels, triple-labeling was imaged in sequential scanning mode.

### Statistics

SigmaStat 3.1 software was used to perform analysis of variance. ANOVA for repeated measures was used for statistical analysis of group effects of electrophysiological data. One- and two-way ANOVA were performed for the analysis of immunohistochemical findings. Results were considered significant when p≤0.05. Pairwise multiple comparison procedures (Scheffé's and Holm-Sidak post-hoc methods for electrophysiology and immunohistochemistry, respectively) were applied to more accurately assess the source of variation between groups.

## Results

### HFS-LTP induces a rapid and sustained elevation of Arc mRNA and protein expression in pre-existing DGCs

HFS in intact, anesthetized rats resulted in rapid and stable potentiation of medial perforant path-evoked field excitatory post-synaptic potentials (fEPSPs) (F_1,55_ = 237.7; p<10^−3^) (Fig. 2a,b). The magnitude and kinetics of LTP observed here were in line with our previous reports and equivalent across all the groups (F_4,55_ = 1.097; p = 0.37) (Fig. 2b). HFS-LTP was associated with a rapid and sustained elevation of Arc mRNA and protein, as demonstrated by in situ hybridization (Fig. 3a,b) and immunohistochemistry (Fig. 3c–e), respectively.

### Time-dependent proliferation, survival and migration of newly generated DGCs

Before further analysis of Arc expression in newborn cells, we first examined the distribution and total number of BrdU-labeled cells in the dentate gyrus (hilus, SGZ and GCL) of young adult male rats which received a single BrdU injection (200 mg/kg) and were sacrificed 1, 7, 14, 21 or 28 days later. BrdU is a thymidine analog which incorporates into the DNA of dividing cells during the S-phase of the cell cycle, rendering it a widely used marker for birth dating dividing cells, monitoring proliferation and determining their fate. HFS-LTP 195 minutes prior to tissue collection had no effect on proliferation and/or survival of BrdU-labeled cells in the hippocampal regions in any age group examined (Hilus: F_1,56_ = 0.5, p = 0.48; GCL: F_1,56_ = 0.248, p = 0.62; SGZ: F_1,56_ = 0.0481, p = 0.83). Data from both hemispheres were thus pooled for further analysis.

A progressive time-dependent reduction in the total number of BrdU-labeled cells was found in the dentate hilus (F_4,28_ = 6.184, p<10^−3^) (Fig. 4c). By day 28, this decline was −53.3% (t = 4.525, p<10^−4^) and −47.2% (t = 3.896, p<10^−4^) compared to days 1 and 7 respectively. In contrast, one-way ANOVA revealed a progressive increase in the GCL (F_4,28_ = 8.141, p<10^−3^) as well as a time effect in the SGZ (F_4,28_ = 16.649, p<10^−3^) (Fig. 4c). Specifically, in the GCL, post-hoc testing revealed a +121% (t = 3.952, p<10^−4^), +161% (t = 5.277, p<10^−5^) and +133% (t = 4.355, p<10^−4^) change in the 7, 14 and 21 day groups compared to day 1, with a respective increase of +57.3% (t = 4.340, p<10^−4^) and +50.0% (t = 3.792, p<10^−4^) in the 7 and 14 day groups compared to day 1 in the SGZ. By 28 days, and in line with expectations, numbers were significantly reduced compared to days 7 (t = 6.991, p<10^−7^), 14 (t = 6.390, p<10^−7^) and 21 (t = 4.765, p<10^−5^) (Fig. 4c).

We also found a significant, time-dependent reduction in the percentage of BrdU+ cells in the SGZ with a corresponding increase in the GCL (F_4,28_ = 6.593, p<10^−3^) (Fig. 4d), suggesting that newly formed cells progressively migrate from the SGZ, where they originate by proliferation of neural stem/neuroprogenitor cells, to the GCL, where they differentiate into granule cell neurons. Specifically, by day 28 the percentage of BrdU-labeled cells in the GCL increased by +145% (t = 4.792, p<10^−5^) and +73% (t = 4.3.745, p<10^−4^) compared to days 1 and 7 respectively. Our results confirm previously established patterns of cell proliferation, survival, and migration during the first month after BrdU injection [29], [30].

### Constitutive expression of Arc in DGCs

**Figure 6:**
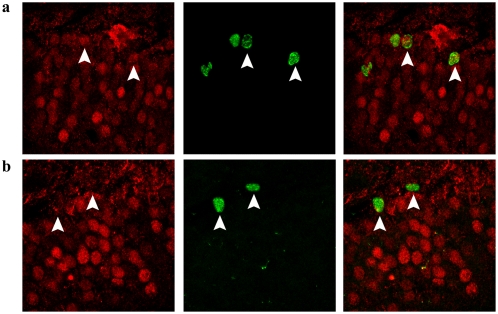
Zif268 expression in newborn granule cells following HFS-LTP. a, Representative photomicrograph, exemplary of the 14–28 day groups, illustrating newborn granule cells positive for BrdU (green) and zif268 (red) expression (white arrows). b, Representative photomicrograph of newborn granule cells negative for zif268 expression (white arrows) as seen in the 1–7 day groups. Zif268 was not spontaneously expressed in new neurons, but could be elicited in 2–4 week old cells in response to LTP induction.

We expected to find Arc expression solely in older neurons which have reached synaptic maturity and are able to undergo long-lasting strengthening of the synapse in response to patterned activation. Immunofluorescent colabeling of BrdU and Arc (Fig. 5a–c) confirmed the expression of Arc in newborn cells. In striking contrast to expectations, however, Arc expression occurred at all ages while no differences between the stimulated and nonstimulated sides was detected in any of the dentate gyrus subregions (Fig. 5d,e). Contrary to their mature counterparts, these young cells failed to exhibit enhanced Arc expression following HFS-LTP, even at 28 days of age. Previous work has shown HFS-LTP-associated expression of zif268 in new neurons of at least 2 weeks of age [31]. We confirmed this report and found that zif268 was not spontaneously expressed in new neurons, but could be elicited in neurons 2–4 weeks of age following LTP induction in the perforant pathway (Fig. 6). The apparently constitutive nature seen here for Arc expression thus appears to be Arc-specific rather than common to all IEGs. Specifically, the ANOVA revealed an effect of time in the GCL (F_41,481_ = 15.716, p<10^−3^) and SGZ (F_41,481_ = 11.227, p<10^−3^), but there were no effects of stimulation or a time-stimulation interaction on BrdU+/Arc+ cells in the hilus (time, F_40,480_ = 1.229, p = 0.311; stimulation, F_10,480_ = 0.0287, p = 0.866; time×stimulation, F_40,480_ = 0.121, p = 0.974), GCL (stimulation, F_10,481_ = 1.399, p = 0.243; time×stimulation, F_40,481_ = 0.445, p = 0.775) or SGZ (stimulation, F_10,481_ = 0.181, p = 0.672; time×stimulation, F_40,481_ = 0.370, p = 0.829) (Fig. 5d,e). Time effects within each hemisphere are presented in the graphs but not described here as the data were pooled and discussed in detail below.

### Arc-positive new cells persist with time, while Arc-negative new cells subside

**Figure 7:**
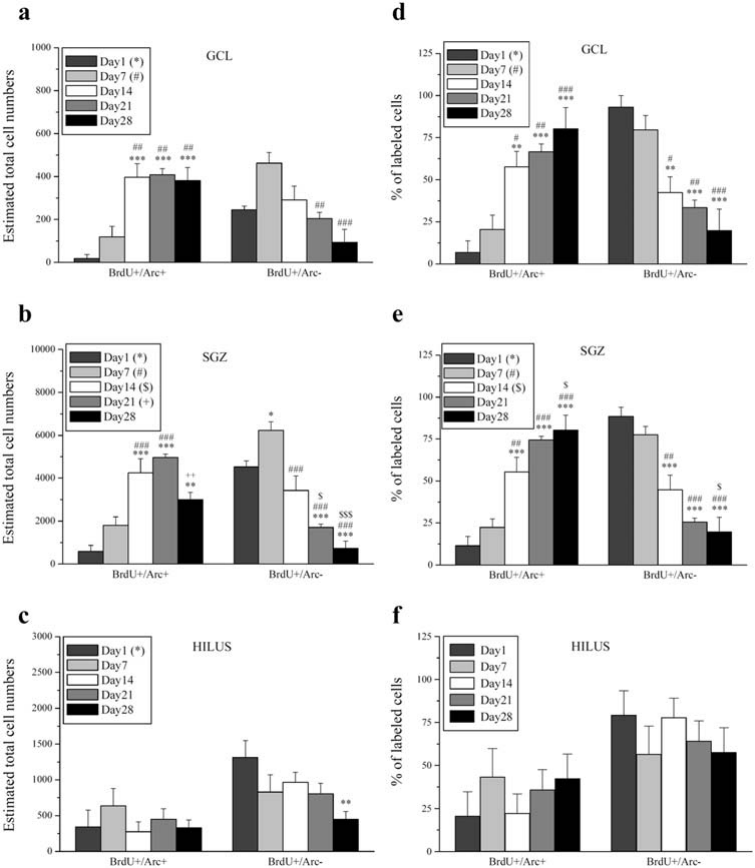
Total BrdU^+^/Arc^+^ cells persist with time while BrdU^+^/Arc^−^ cells subside in the dentate gyrus. a, Graph illustrates the total number of BrdU^+^/Arc^+^ and BrdU^+^/Arc^−^ cells in the GCL. The rise in BrdU+/Arc+ cells in the 14, 21 and 28 day groups compared to days 1 and 7 suggest Arc expression is strongly associated with their survival. b, BrdU^+^/Arc^+^ and BrdU^+^/Arc^−^ cells in the SGZ and c, Hilus represented as total numbers. Total cell numbers represent estimates obtained by multiplying total BrdU cell numbers by respective percentages. d, Number of BrdU^+^/Arc^+^ and BrdU^+^/Arc^−^ cells in the GCL, e, SGZ and f, Hilus depicted as percentages of the total BrdU cell population. Both GCL and SGZ express a similar rise in the percentage of BrdU^+^/Arc^+^ cells coupled to a progressive decline in BrdU^+^/Arc^−^ cells across the ages. The Hilar data show no effects of age but support specificity of the SGZ and GCL findings. The *, #, $, + symbols represents significant effects compared to day 1, 7, 14, 21 respectively. One, two or three symbols represent p<0.05, p<0.005, p<0.0005 respectively.

Given the lack of differences in patterns of BrdU+/Arc+ (Fig. 5d,e) as well as BrdU+/Arc− expression (see File S1), data from the ipsilateral and contralateral dentate gyrus were pooled for further analysis (Fig. 7). Interesting time-dependent effects emerged when the Arc-positive and -negative new cells were compared. In the GCL, the total number of BrdU+/Arc− cells decreased (F_4,22_ = 7.005, p<0.001), while BrdU+/Arc+ cells increased with time (F_4,22_ = 11.468, p<0.001). Specifically, post-hoc analysis revealed a significant increase in BrdU+/Arc+ cells in the GCL in the 14, 21 and 28 day groups compared to days 1 (14: t = 4.941, p<10^−5^; 21: t = 5.104, p<10^−5^ 28: t = 4.747, p<10^−5^) and 7 (14: t = 3.866, p<10^−4^; 21: t = 4.041, p<10^−4^ 28: t = 3.660, p<10^−3^) respectively. BrdU+/Arc− cells in the GCL however, were decreased by 21 (t = 3.589, p<10^−3^) and 28 (t = 5.130, p<10^−5^) days compared to day 7. Taken together, the results demonstrate a correlation between Arc expression, starting in the youngest newborn cells and survival of these cells in the GCL (Fig. 7a). A similar time-dependent effect was also evident in the SGZ, where the increase in BrdU+/Arc+ (F_4,24_ = 17.702, p<10^−3^) was paralleled by a decrease in BrdU+/Arc− cells (F_4,24_ = 28.838, p<10^−3^) (Fig. 7b). Specifically, an increase in BrdU+/Arc+ cells was observed in the 14 and 21 day groups compared to days 1 (14: t = 6.065, p<10^−6^; 21: t = 7.262, p<10^−7^) and 7 (14: t = 4.249, p<10^−4^; 21: t = 5.505, p<10^−5^), while a notable decrease occurred from 21 to 28 days (t = 3.412, p<10^−3^). Although still higher than day 1 (t = 4.009, p<10^−4^), levels at 28 days returned to those comparable with day 7, consistent with cell death and/or migration into the GCL. BrdU+/Arc− cells in the SGZ increased by day 7 (t = 2.844, p<10^−3^) and from there significantly declined by days 14 (t = 4.892, p<10^−5^), 21 (t = 7.89, p<10^−8^) and 28 (t = 9.585, p<10^−9^). Compared to days 1 and 14, levels were significantly reduced by days 21 (1: t = 4.679, p<10^−5^; 14: t = 2998, p<10^−3^) and 28 (1: t = 6.295, p<10^−6^; 14: t = 4.692, p<10^−5^). The hilus was included in all analyses of Arc to obtain a complete representation of the hippocampus, although it did not show any outstanding results. No time effect was found on the number of BrdU+/Arc+ cells (F_4,24_ = 0.669, p = 0.620), and only a slight effect was seen in total BrdU+/Arc− cells (F_4,24_ = 2.909, p = 0.043). Posthoc analysis revealed reduced numbers at 28 days compared to day 1 (t = 3.336, p<10^−3^) as the only significant difference (Fig. 7c).

The BrdU+/Arc+ and BrdU+/Arc− cell populations calculated as percentages of the total new cell population are depicted in figures 7d–f. In the GCL, the percentage of BrdU+/Arc+ cells significantly increased over time (F_4,22_ = 10.508, p<10^−3^). Specifically, post-hoc analysis revealed a significant increase in BrdU+/Arc+ cells in the GCL in the 14, 21 and 28 day groups compared to days 1 (14: t = 3.669, p<10^−3^; 21: t = 4.320, p<10^−4^; 28: t = 5.303, p<10^−5^) and 7 (14: t = 2.867, p<10^−3^; 21: t = 3.561, p<10^−3^; 28: t = 4.608, p<10^−4^) (Fig. 7d). Similarly, a time-dependent effect was evident in the SGZ, where the percentage of BrdU+/Arc+ cells increased gradually over the 28-day period (F_4,24_ = 20.812, p<10^−3^). Post-hoc analysis revealed a significant increase in BrdU+/Arc+ cells in the SGZ in the 14, 21 and 28 day groups compared to days 1 (14: t = 4.526, p<10^−4^; 21: t = 6.501, p<10^−6^; 28: t = 7.118, p<10^−7^) and 7 (14: t = 3.565, p<10^−3^; 21: t = 5.636, p<10^−6^; 28: t = 6.283, p<10^−6^) (Fig. 7e). Again the hilus demonstrated no time effect on the percentage of BrdU+/Arc+ or BrdU+/Arc− cells (F_4,24_ = 0.619, p = 0.653) (Fig. 7f). When presented as percentages in the graphs, statistical significances for BrdU+/Arc− cell populations are shown but not specified here, as the percentages are exactly inversely proportional to those of the BrdU+/Arc+ cells with identical statistical significances. An important note, regards the relatively high percentage of Arc+ new cells reported here, which may appear higher than those seen by some [32], [33]. This could lie in part in our data presentation, as Arc+/− cells are expressed as percentages of the new, BrdU-labeled cells rather than the often used total granule cell population, new and pre-existing. Other differences that could impact the fraction of Arc+ cells are the species used, the animals' age and housing conditions as well as a possible latent effect of BrdU treatment on cell survival [34].

### Arc-positive new cells are neuronal and persist over time

**Figure 8:**
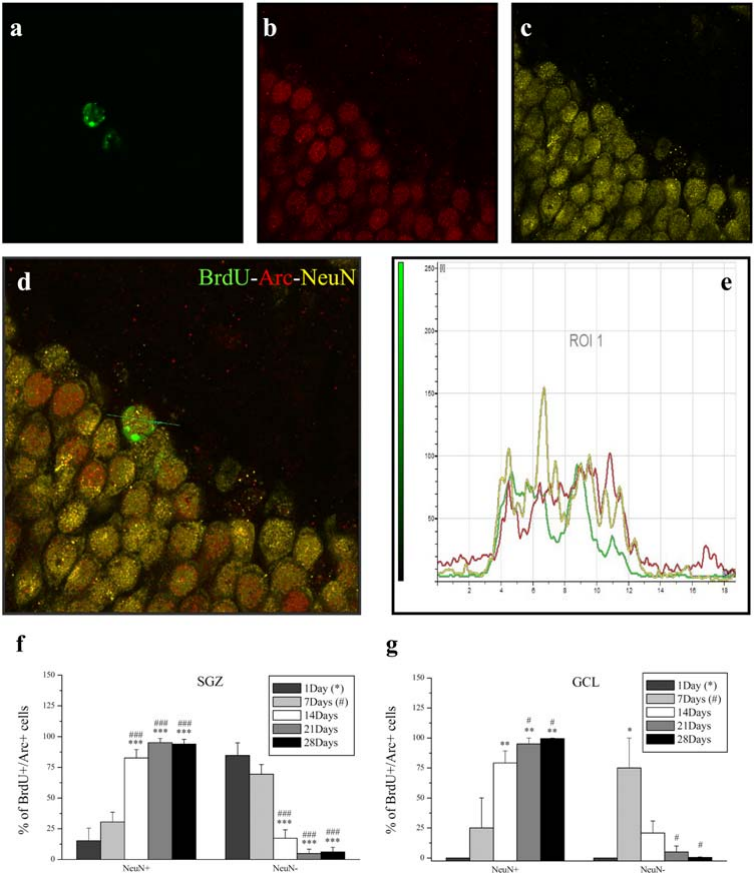
Total BrdU^+^/Arc^+^/NeuN^+^ cells in the dentate gyrus. a, Confocal image of BrdU (green) b, Arc (red) c, NeuN (yellow) d, Merged image illustrating colocalization of BrdU-Arc-NeuN e, Profile measurement of the 3 channels along the indicated line segment verifies colocalization of BrdU-Arc-NeuN in a DGC. Representative images taken from an animal of the 28-day group (195 minutes post HFS). f, Graphs illustrate the percentage of total BrdU+/Arc+ cells which are positive and negative for NeuN in the SGZ and g, GCL. Both regions illustrate a significant and time-dependent increase in NeuN+ cells coupled to a progressive reduction NeuN- indicating that Arc positive new DGCs are neuronal and persist over time. New cells expressed in the GCL by 1 day were negligible. The * and ^#^ symbols represent significant effects compared to days 1 and 7 respectively One, two or three symbols represent p<0.05, p<0.005, p<0.0005 respectively.

Having found that preferential survival seems to occur in newborn cells positive for Arc expression, we proceeded to explore the nature of this specific subpopulation of BrdU cells by performing a triple labeling for BrdU/Arc/NeuN (Fig. 8a–e). The results demonstrate a significant and time-dependent increase in the BrdU+/Arc+/NeuN+ cells (F_4,23_ = 29.395, p<10^−3^) (Fig. 8f) coupled to a progressive reduction in BrdU+/Arc+/NeuN− cells in the SGZ (F_4,23_ = 29.395, p<10^−3^) (Fig. 8g). Post-hoc analysis revealed a significant increase in the number of BrdU+/Arc+/NeuN+ cells by 14, 21 and 28 compared to days 1 (14: t = 6.864, p<10^−7^; 21: t = 7.787, p<10^−8^; 28: t = 8.017, p<10^−8^) and 7 (14: t = 5.567, p<10^−5^; 21: t = 6.577, p<10^−6^; 28: t = 6.776, p<10^−7^), with a corresponding reduction of BrdU+/Arc+/NeuN− cells. Likewise, a similar pattern of expression appeared in the GCL, with a significant time-dependent increase in BrdU+/Arc+/NeuN+ cells (F_4,11_ = 7.761, p<10^−3^) and decrease in BrdU+/Arc+/NeuN− cells (F_4,11_ = 4.443, p = 0.022). Post-hoc analysis revealed a significant increase in BrdU+/Arc+/NeuN+ cells by days 14, 21 and 28 compared to days 1 (14: t = 3.865, p<10^−3^; 21: t = 4.149, p<10^−3^; 28: t = 3.967, p<10^−3^) and 7 (21: t = 3.418, p<10^−3^; 28: t = 3.253, p<10^−3^), while a significant decrease appeared in BrdU+/Arc+/NeuN− cells by days 21 (t = 3.418, p<10^−3^) and 28 (t = 3.253, p<10^−3^) compared to day 7. There were no new cells, positive for Arc, expressed in the GCL after 1 day (irrespective of NeuN expression), giving way to the statistical discrepancy between the otherwise exactly inversely correlated NeuN+ and NeuN− cell populations. Of the entire new DGC population (SGZ and GCL) irrespective of Arc expression, 1.76% (±1.76) assumed the NeuN phenotype by 1 day, 17.6% (±5.6) by 7 days, 62% (±5.7) by 14 days, 74.1% (±5.1) by 21 days and 83.9% (±2.5) by 28 days.

## Discussion

This study examined expression of the immediate early gene Arc/Arg3.1 in dentate gyrus BrdU-labelled cells during the first four weeks of neuronal maturation. Given its pivotal role in late-phase LTP and memory consolidation [19], [20], [23], [35], we surmised that young neurons would not be able to express Arc prior to the formation of the perforant path input and establishment of the postsynaptic machinery underlying induction of Arc, trafficking to synapses, and local protein synthesis-dependent LTP consolidation. By applying HFS, detection of Arc was thus intended as a tool to determine the age at which adult-born DGCs display physiologically equivalent behavior compared to their mature adult counterparts. Three novel findings were obtained: (i) LTP induction within the perforant path does not detectably increase Arc expression in newborn granule cells, suggesting that young neurons are refractory to synaptically-evoked Arc expression (Fig. 5). (ii) Arc is expressed in newly formed cells from a very early post-mitotic age - as early as 1 day after birth (Fig. 5,7,8). (iii) Arc expression is associated with the long-term survival and neuronal differentiation of newly generated cells (Fig. 7,8). These results together with the early post-mitotic expression suggest a novel function for Arc in neurogenesis, distinct from its role in LTP, LTD and homeostatic synaptic plasticity [19], [23], [36], [37].

The dentate gyrus produces new cells throughout adulthood in a variety of mammalian and nonmammalian vertebrates [2]. In young adult rats as many as 9000 cells are generated each day (Fig. 4c) [29]. Although about half of them die within 4 weeks, the majority (approximately 80%) of the remaining cells differentiate into DGCs [38] and survive for at least 6 months in rodents [30]. The developmental stages of maturation are several, each characterized by distinctive physiological and morphological properties indicative of different connectivity levels [39]–[42]. Axonal projections of newborn granule cells reach the CA3 and hilar area 10–11 days after birth to form synapses with their targets. Spine formation occurs approximately 5–6 days later and reaches a peak during the first 3–4 weeks [41]. The timing of axospinous formation on DGCs concurs with electrophysiological studies demonstrating the emergence of glutamatergic synaptic transmission in cells that are 2–3 weeks of age [41], [43], [44]. Newborn cells (type 2 progenitors) initially show depolarizing responses to GABA due to higher intracellular chloride ion concentrations, then undergo a switch to hyperpolarization at 2–4 weeks, coinciding with the onset of dendritic spine formation and glutamatergic responses [40], [45], [46]. By 4 weeks of age, new neurons begin to display the features typical of mature granule cells such as axosomatic, axodendritic and axospinous input, but further modification takes place [39], [41], [42], [44], [47], until about 4 months, when newborn DGCs become physiologically and morphologically indistinguishable from the pre-existing population [48]–[50].

Given this maturational timeline, expression of Arc in 1 and 7 day old cells (Fig. 5d,e and 7), must precede glutamatergic synapse formation onto these cells. This early and seemingly spontaneous expression of Arc has not been observed for other IEGs, including c-fos, zif268, or homer1a [31], [33], [51]. Indeed, previous studies have revealed a developmental time course in the ability to experimentally evoke IEG expression that corresponds to the time of synapse formation at approximately 2 weeks. Kainate- and pentylenetetrazol-evoked seizures induce expression of c-fos, zif268, and Homer1a in neurons at 2–5 weeks, but not earlier [51]. Similarly, LTP induction in the perforant path induces zif268 expression in adult born cells no younger than 2-weeks old [31]. In another study in mice spatial water maze training evoked expression of c-fos in DGCs that were 6 weeks old, but not in 1 week-old BrdU-labeled cells [31], [33], [51]. The spontaneous expression in early newborn cells (less than 4 weeks of age), appears to be a unique property of Arc among the IEGs.

Another intriguing property of new neurons (up to 4 weeks of age) seems to be their refractory nature to HFS-evoked Arc induction. This is all the more surprising when considering that neurons between the ages of 1–3 weeks have lower thresholds for LTP induction relative to pre-existing DGCs [42], [52]. To contrast the unique properties of Arc relative to other IEGs in our paradigm, we sought to replicate the findings of Bruel-Jungerman et al. (2006) regarding zif268 expression in newborn granule cells [31]. In agreement with their report, we found that zif268 was not spontaneously expressed in new neurons, but could be elicited in neurons 2–4 weeks of age in response to LTP induction. The paradigm used here is thus capable of inducing IEG expression in new neurons. However, the lack of HFS-evoked Arc induction does not exclude the possibility that newborn neurons with their own specific synaptic properties, display a form of LTP independent of Arc expression. Intracellular recording of LTP and quantitative measurements of Arc mRNA levels in single granule cells are needed to resolve the issue. Newborn neurons might show dendritic development and integration very early after BrdU incorporation but largely independent of LTP as they have not yet reached the layers where perforant path input would affect them. Future experiments designed to examine a role of Arc in adult neurogenesis exerted by non-HFS factors (i.e. GABA, serotonin or glutamate from non-perforant path sources) would be of interest with regard to the current data.

Ramirez-Amaya et al. (2006) have recently demonstrated enhanced behaviourally-evoked expression of Arc in 5 month old DGCs relative to the older, pre-existing neurons [32]. Kee et examined recruitment of newborn neurons into dentate circuitry following spatial training performed between 1 and 8 weeks post-BrdU injection [33]. Recruitment was based on the expression of c-fos and Arc following a probe trial performed at 10 weeks after BrdU injection. That study demonstrated enhanced IEG expression in newborn DGCs relative to pre-existing neurons in mice trained between 4 to 8 weeks after BrdU-labelling. More important for the present discussion, the work demonstrates behavioural induction of Arc in 10 week-old DGCs. Taken together with the present data, this suggests that neurons undergo an early refractory period to evoked Arc expression which gives way to a state of heightened sensitivity as the neurons mature. However, systematic comparisons of behaviourally-evoked and HFS-evoked expression of Arc and other IEGs across the full time-frame of DGC maturation are needed to validate the hypothesis.

In the current study, a relatively large fraction of newborn cells express Arc at 1 and 7 days (Fig. 7; ∼500 cells/dentate gyrus at 1 day; ∼2000 cells/dentate gyrus at 7 days). Previous data suggests that Arc, similarly to c-fos and zif268, is expressed only by cells which have already acquired a neuronal phenotype. It remains to be determined, however, whether Arc expression is restricted to newborn cells with a neuronal fate or also occurs in proliferating cells prior to fate determination. To this end it would be interesting to know whether these 1 and 7 day old BrdU+/Arc+ cells are still proliferating. Regulation of adult hippocampal neurogenesis occurs primarily during stages associated with doublecortin (DCX) expression [45]. This protein is transiently expressed during a period extending from proliferative progenitors to post-mitotic neurons with extended dendrites. Kempermann and colleagues determined that ∼20% of DCX positive cells are still in cell cycle while 70% are post-mitotic. This implies that the remaining 10% of DCX positive cells are in progression from a progenitor to an early postmitotic stage (late neuroblasts/immature neurons) [53]. Since at any given time more than 20000 cells express DCX in the dentate gyrus of young adult rats [54], the relatively high number of BrdU+/Arc+ cells found at 1 day might still be DCX positive cells that were cycling at the time of BrdU administration but became post-mitotic during this 24-hour interval. In future studies it will be important to examine the possibility that the BrdU+/Arc+ cells at day 1 correspond to a population of DCX-positive late neuroblasts or immature neurons.

The present study demonstrated a progressive increase in double BrdU/Arc positive cells, paralleled by the gradual time-dependent decline of Arc-negative new DGCs (Fig. 7). By 4 weeks of age, almost the entire (99%) BrdU/Arc-DGC population had become neuronal in nature (Fig. 8g) which also held true for the SGZ where 95% of Arc cells had undergone a neuronal fate (Fig. 8f). Apparently the older they get, the more cells express Arc, which raises an important question. Do newborn cells survive and mature because they express Arc or do they express more Arc as they mature? The fact that essentially all new granule cells express Arc (in contrast to i.e. c-fos or zif268) suggests that at some point in their life all newborn neurons will express this protein. This could account for the expression seen in older newborn cells (such as the 2-, 3- and 4-week old cells), but does not address whether Arc affects the survival of younger cells (24-hour and 1-week old cells). Our data clearly raises the possibility that early Arc expression defines a subpopulation of newborn DGCs with the highest probability of survival and incorporation into the pre-existing hippocampal circuit. Further studies involving selective manipulation of Arc expression in progenitor populations and newborn neurons are needed to establish a causal relationship.

The mechanisms connecting Arc expression to neurogenesis will be important to explore. At the moment two candidate mechanisms stand out, depending on the age of the neurons. In undifferentiated cells, prior to synapse formation, Arc could act directly in the nucleus to promote proliferation, differentiation and survival. Arc accumulates in the nucleus of hippocampal neurons where it localizes to promyelocytic leukemia nuclear bodies (PML-NBs) [55], [56]. PML-NBs are dynamic and heterogeneous protein complexes that vary in number and size depending on the cell type, cell cycle phase, and stage of differentiation [57]. PML-NBs have been implicated in nuclear events such as transcription, heterochromatin formation, post-translational modifications [58]–[60] and cellular functions such as proliferation, apoptosis and senescence [57], [59], [61]. In older DGCs forming excitatory synapses, NMDA receptor activation and LTP induction is known to promote long-term survival [31], [62]. At this later stage, Arc might promote survival through its critical function in consolidating LTP [19].

## Supporting Information

File S1 LTP had no effect on BrdU+/Arc− expression. Besides a time effect in the SGZ (F44,481 = 60.898, p<0.001), there were no effects of time, stimulation or their interaction on BrdU+/Arc− cells in the hilus (time, F40,480 = 0.589, p = 0.672; stimulation, F10,480 = 0.008, p = 0.929; time×stimulation, F40,480 = 0.162, p = 0.957), GCL (time, F40,480 = 1.040, p = 0.397; stimulation, F10,480 = 1.070, p = 0.306; time×stimulation, F40,480 = 0.607, p = 0.659) or SGZ (stimulation, F10,481 = 0.161, p = 0.690; time×stimulation, F40,481 = 1.054, p = 0.390). Post-hoc analysis revealed that compared to days 1 and 7, BrdU+/Arc− cell numbers in both hemispheres were significantly reduced by days 14, 21 and 28 with a further decrease from day 14 to 21 and 28 on the stimulated side. The *, #, $ symbols represent significant effects compared to days 1, 7, and 14 respectively. One, two or three symbols represent p<0.05, p<0.005, p<0.0005 respectively.
(0.13 MB TIF)
