# Supplementary figures and images for: Selective Survival and Maturation of Adult-Born Dentate Granule Cells Expressing the Immediate Early Gene Arc/Arg3.1
Source: PLoS One. 2009 Mar 17;4(3):e4885. doi: 10.1371/journal.pone.0004885 (PMC2654102; doi:10.1371/journal.pone.0004885)

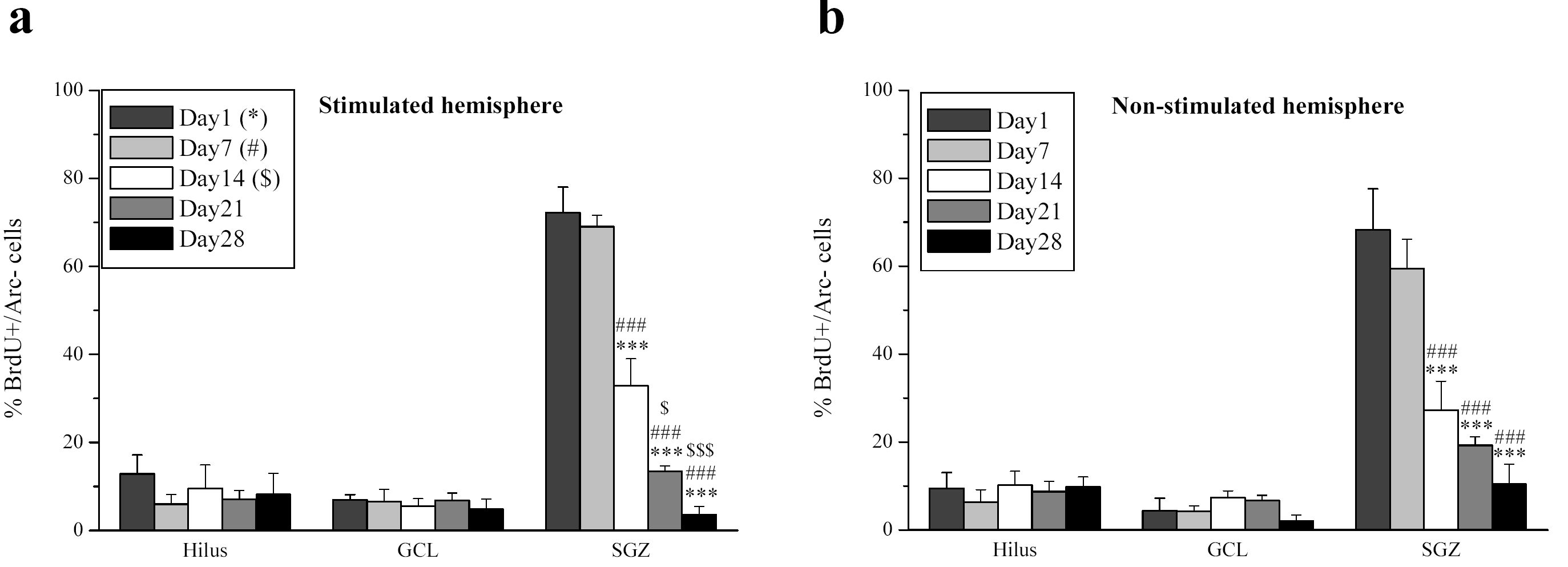

Supplement: File S1 — LTP had no effect on BrdU+/Arc− expression. Besides a time effect in the SGZ (F44,481 = 60.898, p<0.001), there were no effects of time, stimulation or their interaction on BrdU+/Arc− cells in the hilus (time, F40,480 = 0.589, p = 0.672; stimulation, F10,480 = 0.008, p = 0.929; time×stimulation, F40,480 = 0.162, p = 0.957), GCL (time, F40,480 = 1.040, p = 0.397; stimulation, F10,480 = 1.070, p = 0.306; time×stimulation, F40,480 = 0.607, p = 0.659) or SGZ (stimulation, F10,481 = 0.161, p = 0.690; time×stimulation, F40,481 = 1.054, p = 0.390). Post-hoc analysis revealed that compared to days 1 and 7, BrdU+/Arc− cell numbers in both hemispheres were significantly reduced by days 14, 21 and 28 with a further decrease from day 14 to 21 and 28 on the stimulated side. The *, #, $ symbols represent significant effects compared to days 1, 7, and 14 respectively. One, two or three symbols represent p<0.05, p<0.005, p<0.0005 respectively. (0.13 MB TIF) [file pone.0004885.s001.tif]
